# Differential Regulation of TFEB-Induced Autophagy during Mtb Infection and Starvation

**DOI:** 10.3390/microorganisms11122944

**Published:** 2023-12-08

**Authors:** Richa Dwivedi, Piyush Baindara

**Affiliations:** 1 Department of Pathology, University of Pittsburgh, Pittsburgh, PA 15260, USA; 2 Radiation Oncology, NextGen Precision Health, School of Medicine, University of Missouri, Columbia, MO 65211, USA

**Keywords:** autophagy, *Mycobacterium tuberculosis*, TFEB, infection, starvation, host-directed therapies

## Abstract

Through the promotion of phagolysosome formation, autophagy has emerged as a crucial mechanism to eradicate intracellular *Mycobacterium tuberculosis* (Mtb). A cell-autonomous host defense mechanism called lysosome biogenesis and autophagy transports cytoplasmic cargos and bacterial phagosomes to lysosomes for destruction during infection. Similar occurrences occurred in stressful or starvation circumstances and led to autophagy, which is harmful to the cell. It is interesting to note that under both hunger and infection states, the transcription factor EB (TFEB) acts as a master regulator of lysosomal activities and autophagy. This review highlighted recent research on the multitier regulation of TFEB-induced autophagy by a variety of host effectors and Mtb sulfolipid during Mtb infection and starvation. In general, the research presented here sheds light on how lysosome biogenesis and autophagy are differentially regulated by the TFEB during Mtb infection and starvation.

## 1. Introduction

Mtb is an intracellular bacterium that may avoid fusion with phagolysosomes to live and stay within macrophages [1,2]. According to the World Health Organization’s global tuberculosis report, 2022, 10.6 million people contracted the disease, and 1.6 million people are predicted to have died from it in just the year 2021 (https://www.who.int/publications/i/item/9789240061729, accessed on 6 October 2023). Numerous methods are employed by host macrophages to eliminate pathogenic mycobacteria [1]. As a result of multiple extracellular and intracellular challenges, including food or growth factor deficiency, oxidative stress, an accumulation of damaged organelles or misfolded proteins, and viral or microbial infection, Mtb is removed from infected macrophages through the onset of autophagy. Autophagy controls the intracellular loads of Mtb in macrophages as a result; however, Mtb evolved several immunological escape strategies to promote infection [3].

One of the transcription factors involved in the induction of autophagy is TFEB, which is a crucial regulator of autophagic activation [4,5]. The TFEB belongs to the family of proteins known as the microphthalmia/transcription factor E (MiT/TFE) and is a basic helix-loop-helix-leucine-zipper (bHLH-Zip) protein [5]. The TFEB, transcription factor E3 (TFE3), transcription factor EC (TFEC), and melanocyte inducing transcription factor (MITF) are the four members of the MiT-TFE family. The same basic domain that binds particular DNA sequences, a helix-loop-helix (HLH), and a leucine-zipper (Zip) region that is crucial for protein-protein interactions with other MiTF/TFE family members is shared by all four of the members [5,6]. A conserved activation region is also present in TFEB, MITF, and TFE3 and is crucial for the transcriptional activation of these genes [7]. The most divergent member of the family, TFEC, lacks the activation domain and appears to inhibit rather than trigger transcription [6]. Several genes, including those involved in substrate targeting, degradation, and autophagosomal and lysosomal biogenesis, are transcriptionally regulated by the TFEB. By attaching to a 10-base E-box-like motif at the promoter region known as coordinated lysosomal expression and regulation (CLEAR), the TFEB also stimulates the transcription of several lysosomal genes. Through post-translational changes, protein-protein interactions, and spatial organization, the TFEB function is tightly controlled. In resting cells and nutrient-rich environments, the TFEB is primarily cytosolic and inactive [8,9]. However, the TFEB quickly moves to the nucleus in response to hunger or lysosomal dysfunction and triggers the transcription of its target genes. The degree of phosphorylation of the TFEB is a major determinant of both its cellular location and activity. Ser142 and Ser211, two specific serine residues in the TFEB protein, are critical regulators of the protein’s subcellular distribution [10]. The TFEB is kept in the cytoplasm when both of these two serine residues are phosphorylated. It has been demonstrated that phosphorylation of Ser211 in particular acts as a docking site for the chaperone 14-3-3, which locks the TFEB in the cytoplasm and inhibits it from moving to the nucleus, most likely by obscuring its nuclear localization signal (NLS) [11]. Additionally, the TFEB positively controls the expression of genes in the CLEAR network, including ATG9, LC3, SQSTM1, and LAMP1, which are engaged in multiple sequential phases of autophagy in response to famine, stress, or lysosomal dysfunction [10,12,13,14]. It is discovered through starvation tests that the TFEB controls its transcription by interacting with the CLEAR sites in the first intron [15]. Other transcriptional regulators of the TFEB include the fasting transcriptional activator cAMP response element-binding protein (CREB) and the fed state sensing nuclear receptor farnesoid X receptor (FXR), which respectively inhibit or induce the TFEB expression in the liver when in a fed or fasted state. The FXR-CREB axis performs as a crucial physiological switch that controls the TFEB-mediated autophagy, leading to continuous autophagic regulation of nutrients during feeding and fasting cycles [16]. Then, in nutrient-rich conditions, the mTORC1 phosphorylates TFEB on lysosomes, where it remains in the cytoplasm. The TFEB is dephosphorylated and translocates from the cytoplasm to the nucleus in response to starvation, where it upregulates genes related to autophagy and lysosomal biogenesis [17,18]. In this review, we discussed new developments about the direct or indirect induction of autophagy by various host effectors via nuclear translocation of the TFEB, a key transcriptional regulator of the autophagy-lysosomal pathway genes to eradicate Mtb that is still present in macrophages. We paid particular attention to the differential regulation of the TFEB-induced autophagy during Mtb infection and starvation along with the possible target for the development of host-directed therapies (HDTs) against Mtb.

## 2. TFEB Is Differentially Regulated by Host Factors to Restrict Mtb Growth

Several host factors, including PPAR (nuclear receptor peroxisome proliferator-activated receptor), NR1D1 (nuclear receptor subfamily 1, group D, member 1), IFNγ, IRGM (Immunity related GTPase M), TRIMs (tripartite motif family proteins), and NCoR1 (Nuclear receptor corepressor) mediate the nuclear translocation of TFEB, a significant transcription factor that connects autophagy and lysosome formation, as a result of Mtb infection.

**Figure 1:**
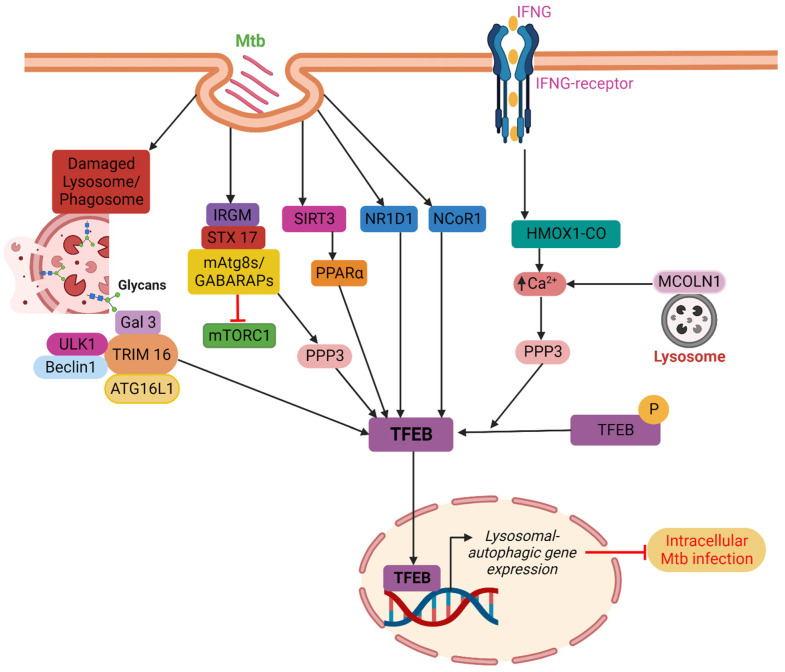
TFEB localization into the nucleus is indirectly influenced by PPARα, IFNγ, IRGM, and TRIMs. PPARα facilitates the disposal of Mtb in a TFEB-dependent manner through the SIRT3–PPARα–TFEB axis. IFNγ stimulates autophagy and Mtb clearance via the PPP3-TFEB signaling axis in a way that is reliant on HMOX1. IRGM blocks mTORC1 while encouraging PPP3 activity, which together results in efficient TFEB activation. TRIM16 binds ATG16L1 and is associated with the key autophagy regulators ULK1 and Beclin1. Furthermore, TRIM16 controls the activity of mTORC1 and the localization of TFEB in the nucleus and cytoplasm. Host factors such as NR1D1 and NCoR1 directly promote the nuclear localization of TFEB. Finally, TFEB causes lysosomal biogenesis and boosts autophagic flow in the nucleus that promotes the clearance of intracellular Mtb from infected macrophages.

***PPAR****:* Kim et al. have demonstrated that the PPAR is essential for eliciting a host response during Mtb infection. The PPAR controls inflammation, mitochondrial and peroxisomal activity, and energy metabolism. Additionally, the PPAR activates the TFEB and prevents the development of lipid bodies. During Mtb infection, the TFEB silencing decreases phagosomal maturation and antibacterial responses while increasing macrophage inflammatory responses. According to this work, the TFEB is necessary for Mtb clearance and the PPAR-mediated activation of the autophagy-lysosomal pathway [19]. Later in 2019, Kim et al. demonstrated that the mitochondrial deacetylase sirtuin 3 (SIRT3) can lessen oxidative stress and pathological inflammation following Mtb or *Bacillus Calmette*-Guerin (BCG) infection. The SIRT3’s anti-mycobacterial activity is carried out via PPAR and TFEB. It is proven that mice lacking Sirt3 had higher bacterial loads and more lung inflammation than mice with Sirt3, although macrophages lacking Sirt3 were able to regain their anti-mycobacterial activity by overexpressing either TFEB or PPAR. Overall, it can be said that the SIRT3-PPAR-TFEB axis is crucial in promoting host resistance against Mtb infection by antibacterial autophagy (Figure 1) [20].

***NR1D1****:* Several cell types, including adipose, vascular smooth muscle, skeletal muscle, liver, heart, brain, and immune cells like T cells and macrophages, express NR1D1, which controls their functions [21]. According to reports, the NR1D1 participates in the destruction of Mtb by triggering the autophagy and lysosomal biogenesis pathways, and its overexpression is linked to an increase in the production of the TFEB. This finding shows that the NR1D1 stimulates autophagy through the TFEB in conjunction with lysosome biogenesis to enhance Mtb clearance (Figure 1) [22].

***IFNγ****:* According to research, Mtb is removed by interferon-gamma (IFNγ)-induced autophagy. In response to the immunomodulation by IFNγ, autophagy is induced and Mtb living in macrophages is killed with the help of carbon monoxide (CO) produced by heme oxygenase 1 (HMOX1). Through the lysosomal Ca^2+^ transporter MCOLN1/TRPML1, which is activated by the HMOX1-produced CO, the cytosolic Ca^2+^ levels are raised. This dephosphorylation of the TFEB leads to an increase in autophagy and lysosomal biogenesis. These results demonstrate that IFNγ induces autophagy and Mtb clearance via the PPP3-TFEB signaling axis in an HMOX1-dependent manner (Figure 1) [23]. Furthermore, the TRPML1 might indirectly or directly facilitate the actions of calcineurin on the TFEB through endoplasmic reticulum stress and reactive oxygen species (ROS) [24]. According to studies, TRPML1 depletion prevents the lysosomal Ca^2+^ release and calcineurin activation, which prevents the TFEB activation and the induction of autophagy in response to food deprivation [25]. A separate study proposes TRPML1 as a lysosomal membrane-based ROS sensor that controls an autophagy-dependent negative feedback loop to alleviate cellular oxidative stress [26].

***IRGM:*** Immune-related another host component that aids in resistance to Mtb infection is the IRGM protein family. The human IRGM polymorphisms are linked to differential susceptibility to mycobacterial illness, and they also facilitate the delivery of Mtb into autolysosomes that degrade proteins [27,28]. The TFEB activation, downstream autophagy, and lysosome biogenesis are primarily mediated by the IRGM. Gamma-aminobutyric acid receptor-associated protein (GABARAP), LC3, other mammalian Atg8 proteins (mAtg8s), other ATG proteins, and the SNARE protein STX17 interact with IRGM to cause the development of autolysosomes. The IRGM, on the other hand, blocks mTORC1 while encouraging PPP3 activity, which together results in efficient TFEB activation. The mAtg8s regulate TFEB, a crucial regulator of lysosomal biogenesis, while, the autolysosomal route is controlled by the IRGM, STC17, and mAtg8s via TFEB activation (Figure 1) [29].

***TRIMs****:* Galectins, which are important in selective autophagy, interact with TRIMs on a large scale [30]. In 2016, Chauhan et al. demonstrated that TRIM16 identifies endomembrane damage through interactions with Galectin-3 in a ULK1-dependent way using lysosomal and phagosomal damage models. In addition to being connected to important autophagy regulators ULK1 and Beclin1, TRIM16 binds ATG16L1. Additionally, TRIM16 controls the activity of mTORC1 and the localization of TFEB in the nucleus and cytoplasm. In conclusion, TRIM16, ATG16L1, and Galectin-3 are necessary for Mtb clearance via TFEB (Figure 1) [31].

***NCoR1****:* Using the AMPK-mTOR-TFEB signaling axis, the NCoR1 mediates auto-phagolysosomal pathway fine-tuning in Mtb pathogenesis regulation. The significance of NCoR1 for the regulation of host defense against infection is highlighted by its increased expression in myeloid cells during the initial stages of Mtb infection. By adjusting the AMPK-mTOR signaling axis, which in turn controls the TFEB activity, NCoR1 loss of function hinders the clearance of Mtb H37Rv and *M. smegmatis* infection in myeloid cells. Mtb benefits from a survival advantage when the NCoR1 is reduced since the TFEB independently regulates lysosomal biogenesis and autophagic machinery. The bacterial burden is eliminated by the overexpression of the TFEB in NCoR1 deficient macrophages, which also restored the expression of LC3 and LAMP1. This study summarizes that the NCoR1 directly regulates Mtb survival by preserving the ideal auto-phagolysosomal pathway. Additionally, their findings demonstrated a strong connection between them by revealing a clinically significant association of NCoR1 expression during active TB infection, which is recovered after 12 months of TB therapy. Comparable results are seen in the Mtb-infected PBMCs, wherein the NCoR1 expression is elevated at the beginning of infection and then reduced after 24 h. In terms of treatment, NCoR1 may be a good fit for HDTs (Figure 1) [32].

Overall, the TFEB is controlled differently under strict multitier regulation of several host variables to control the intracellular Mtb growth. Therefore, the TFEB-induced autophagy regulatory mechanisms that are cross-intervened during infection and starvation might be developed as potential HDTs to combat tuberculosis. However, on the other hand, Mtb can also inhibit autophagy via restricting acidification and phagolysosome maturation, along with autophagosome fusion to the lysosomes [1,33]. Therefore, further detailed studies are required for a complete understanding of Mtb pathogenesis.

## 3. Regulation of the mTORC1-TFEB Axis and Mtb Clearance under Starvation

**Figure 2:**
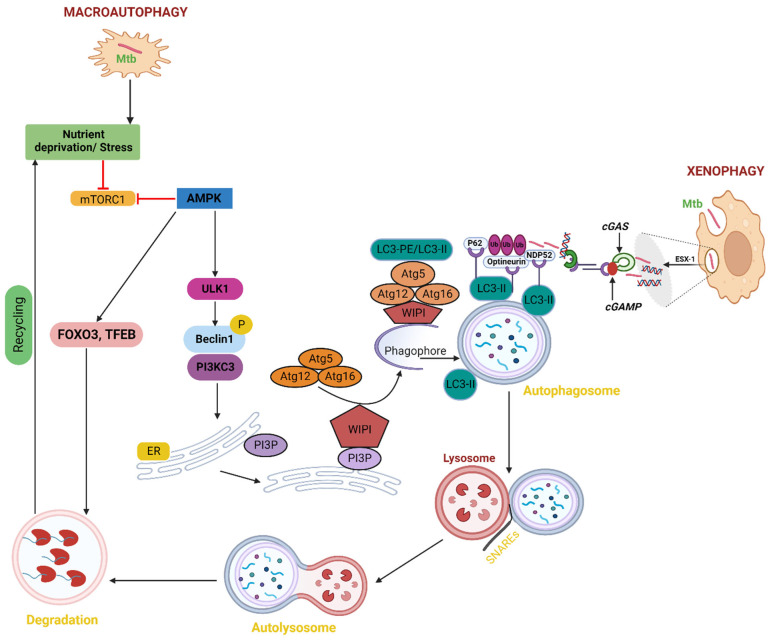
Macroautophagy and autophagy caused by starvation in Mtb. Starvation inhibits mTORC1, which in turn triggers ULK1 activation, the creation of autophagosomes, and TFEB activation, which further boosts the transcription of genes related to autophagy and lysosomes. Phagophores combine to generate autophagosomes. The phagophore enlarges by capturing cytoplasmic cargos, and Mtb contracts to create an autophagosome, a double-membrane vesicle. The Mtb and cytoplasmic cargos are then quickly broken down by lysosomal hydrolases and released into the cytosol for additional recycling after the lysosome and autophagosome combine to produce autolysosomes. Pathogens inside cells are targeted by xenophagy for lysosomal breakdown. The same processes that are involved in classical autophagy; initiation, elongation, substrate targeting, maturation/lysosomal fusion, which destroys cargo are also present in xenophagy. In the case of Mtb infection, substrate targeting entails the ubiquitination of bacteria or the colocalization of ubiquitin to bacteria. The recognition of the ubiquitin by autophagy receptors that interact with LC3 to attract the bacteria to autophagosomes, which then fuse to lysosomes to degrade the cargo, is then necessary for the recruitment of the bacteria to the autophagosomes.

Inducing autophagy through starvation can destroy pathogenic Mtb in macrophages by simulating stress or infection conditions [34]. Induced by nutritional deprivation, cellular injury, or stress, starvation-induced autophagy, also known as macroautophagy, involves the breakdown of damaged organelles, abnormally folded or folded proteins, and cytoplasmic foreign entities. By engulfing bacteria in autophagosomes, which fuse with lysosomes to produce autolysosomes, pathogens are destroyed in a process known as xenophagy [35,36]. To liberate nutrients for the de novo production of molecules during starvation, autophagy sequesters the cytosolic components of cells [37]. Unc-51-like kinase complex (ULK1) dissociates from adenosine monophosphate-activated protein kinase (AMPK) during stress situations such as nutrient deprivation or Mtb invasion. Beclin 1 is phosphorylated by the ULK1 complex, which then joins with phosphatidylinositol 3-kinase class 3 (PI3KC3) to create a complex. Phosphatidylinositol 3-phosphate (PI3P) is produced on endoplasmic reticulum membranes by beclin1-PI3KC3 complexes. The creation of an autophagic phagophore, which develops into a whole autophagosome, is then signaled by PI3P [38]. The WD-repeat protein interacting with phosphoinositide (WIPI) is recruited by the synthesis of PI3P to aid in the development of the phagophore. The ubiquitin-like conjugation mechanism, which is unique to autophagy, is recruited to the phagophore by WIPI2B. The lipidation of microtubule-associated protein 1A/1B light chain 3 (LC3) to phosphatidylethanolamine, also known as LC3 lipidation, is facilitated by WIPI2B-dependent recruitment of the ATG12-5/ATG16 complex. Additionally, LC3 lipidation causes the double membrane to self-fuse to form the autophagosome, which then merges with the lysosome to break down the ingested contents [39]. Soluble N-ethylmaleimide-sensitive factor attachment (SNARE) Syntaxin 17 (STX 17) promotes this fusion of the autophagosome and lysosome, and trapped Mtb or other cellular cargos are ultimately broken down in the resulting autolysosomes [38]. This is accomplished by increasing the expression of genes related to autophagy and lysosomal biogenesis by the AMPK-activated transcription factors Forkhead Box O3 (FoxO3) and TFEB (Figure 2) [40,41].

It is known that several bacterial effector proteins can influence autophagy. The type I to type VII and type IX secretion systems are used to secrete several of these effectors [42]. Numerous Type VII secretion systems (Esx1–Esx5) are present in mycobacteria. Mycobacterial egress is made possible by the phagosome being punctured by Mtb ESX-1 [43]. Cyclic GMP-AMP synthase (cGAS), a cytosolic DNA sensor that detects mycobacterial DNA, releases cyclic guanosine monophosphate (cGMP) as soon as it detects mycobacterial DNA during Mtb infection [44]. Numerous of these effectors are produced by type I to type VII and type IX cGMP, which is recognized by the stimulator of interferon genes (STING) and causes the production of type I IFN as well as the recruitment of the autophagy receptors p62, NDP52, and optineurin [45,46]. These receptors are attracted to the ubiquitinated pathogen, enabling the autophagosome to target it precisely. To bind the LC3 autophagy protein, the receptors have an LC3 interaction region (LIR) (Figure 2) [47,48]. According to studies, the pathogenic Mtb H37Rv is suppressed when autophagy is induced in infected macrophages through fasting or rapamycin [34]. This effect is reversed by conventional autophagy inhibitors. Phagosomes carrying mycobacteria associate with Beclin 1, a hVPS34 PI3K component that promotes autophagy [49,50]. Therefore, it is likely that mycobacterial phagosomes recruit Beclin 1 complexed with PI3-kinase when there is hunger. In cells induced for autophagy, phagosome colocalization with Beclin 1 exceeds 60% of all mycobacteria-containing vacuoles, roughly matching the total decline in mycobacterial viability. Studies have shown that mutation or knockdown of host genes linked with autophagy, such as p62, Beclin1, Atg5, Atg7, or Unc-51-like kinase 1 (Ulk1), increases the survival of intracellular bacteria [51,52]. In general, the mTORC1-TFEB axis plays a significant role in controlling autophagy under starvation and stress circumstances. Starvation and Mtb-induced autophagy appear to be tightly regulated by many levels of the mTORC1-TFEB axis, and they may be new targets for the development of anti-Mtb drugs. However, one should not forget that Mtb can also inhibit autophagy as its own immune escape strategies.

## 4. Induction of Autophagy as a Host-Directed Therapeutic Approach against Mtb

Mtb can persist in a dormant, semi-replicating, or non-replicating form in the granuloma environment. It has been reported that granulomas help the host by preventing Mtb from spreading to other tissues or organs by enclosing the affected region with activating immune cells [53]. Also, the host-pathogen interactions in granulomas are extremely complicated, with the potential for bacterial death as well as survival. The host immunological response and the bacterial capacity to withstand or evade it are considered to have a cumulative influence on the intracellular survival of Mtb [54]. Thus, enhancing the immune repertoire to battle Mtb by the HDTs may be one strategy for efficient clearance and bacterial death [55]. To maximize the pro-inflammatory response or alter tissue physiology, host-directed treatment attempts to manipulate the metabolism and/or immune cell activity [56,57,58]. Due to the potential to repurpose medications that have already received approval for the treatment of chronic illnesses and the benefit that pathogenic bacteria, like Mtb, cannot become resistant to an HDT because it targets host cell functions, research on HDT as a potential therapeutic strategy for infectious diseases has recently gathered significant strength [59,60]. Thus, one emerging idea in the treatment of several chronic illnesses is to target the autophagy mechanism using small molecules and medications to enhance the host cell effector functions [61,62]. Here are the details of some known autophagy inducers that combat Mtb infection via upregulation of the TFEB.

***GSK4112***: It has been shown that the synthetic small molecule GSK4112 increases lysosome biogenesis and induces autophagic flow by acting as an agonist to NR1D1 [22]. TFEB expression is modulated by the activation of the transcriptional protein NR1D1, which is important in infection and inflammation [63]. When Mtb strain H37Rv infected THP-1 cells, the overexpression of the TFEB increased the quantity of both autophagosomes and lysosomes [22].

***GW7647***: A synthetic small molecule agonist of the PPARα transcription factor, GW7647, has been shown by Kim et al. to increase autophagic flow in mice BMDMs against *M. bovis* BCG and Mtb strain H37Rv. The overexpression and translocation of TFEB, a crucial regulator of many genes involved in autophagic flux, is the outcome of GW7647’s activation of PPARα. Moreover, during mycobacterial infection, PPAR-α activation prevented the development of lipid bodies [19].

***Lipid-lowering drugs (Simvastatin, Pravastatin, Rosuvastatin, Atorvastatin)***: These drugs decrease the Mtb load in human macrophages by lowering cholesterol levels and modulating the AMPK-mTORC1-TFEB axis in ways that encourage autophagy for Mtb clearance along with phagosome maturation and lysosome fusion [64,65].

***Wy14643***: It activates the PPARα receptor protein and subsequently enhances autophagic flux via upregulation of the TFEB signaling, along with increased lipid catabolism [19].

***Compound 2062***: An aminopyrimidine with the chemical name 2062 was the subject of a different investigation by Bryk et al. in 2020, which demonstrated enhanced control of intracellular Mtb in conjunction with rifampicin and linked with the activation of TFEB, which encourages lysosomal activation to accelerate Mtb clearance. According to this study, specific TFEB activators can enhance host control of Mtb infection and enhance the effectiveness of low-dose rifampin [66].

***Ambroxol***: This mucoactive drug suppresses excessive mucus secretion by inhibiting NO-dependent activation of soluble guanylate cyclase that further induces autophagy via TFEB nuclear translocation stimulation [67].

***Honokiol***: Low molecular weight polyphenol honokiol has been shown to enhance the autophagic activities in human peripheral blood mononuclear cells (PBMCs), BMDMs, and HMDMs against strains of *M. bovis* BCG and Mtb H37Rv. It does this by activating SIRT3 deacetylase. The activation of SIRT3 by honokiol-mediated autophagy led to the subsequent induction of PPARα transcription factor expression, which in turn caused the overexpression and translocation of the TFEB, a crucial regulator of several genes involved in autophagic flux [20].

***Bedaquiline***: Bedaquiline sparks autophagy to promote clearance of Mtb. It induces autophagy by upregulating lysosomal activation via the TFEB and calcium signaling and potentiates the exertion of other anti-TB medicines [68].

***Trehalose***: Naturally occurring disaccharide trehalose has been shown to promote autophagy in many cell lines to combat Mtb and non-tuberculous mycobacterial (NTMs) [69]. Trehalose elevated phosphatidylinositol 3,5-bisphosphate, which functioned as an agonist for the mucolipin subfamily, member 1 (MCOLN1) channel and enhanced the release of Ca^2+^ from the lysosomal lumen, therefore inducing autophagic flow [70]. Released Ca^2+^ causes calcineurin, a serine-threonine phosphatase, to become active. This dephosphorylates TFEB and causes trehalose-mediated nuclear translocation of TFEB, which in turn triggers mTOR-independent autophagy in macrophages. Furthermore, trehalose competitively inhibited the GLUT transporters SLC2A3/GLUT3 and SLC2A8/GLUT8, inducing autophagy through AMPK activation and mTOR inhibition that resulted in a response that is similar to pseudo-starvation [69].

Autophagy targeting HDTs is an appealing adjunct to current medications. Importantly, autophagy inducers can restrict the Mtb growth via induction of TFEB-induced autophagy as well as can also combat the autophagy inhibition by Mtb itself. However, before being tested as a therapeutic intervention for tuberculosis in human clinical trials, considerable pre-clinical research employing suitable animal models of tuberculosis is necessary to determine the efficacy, toxicity, and other characteristics of these HDTs.

## 5. Mtb Sulfolipid Controls the mTORC1-TFEB Axis and Prevents Infection

**Figure 3:**
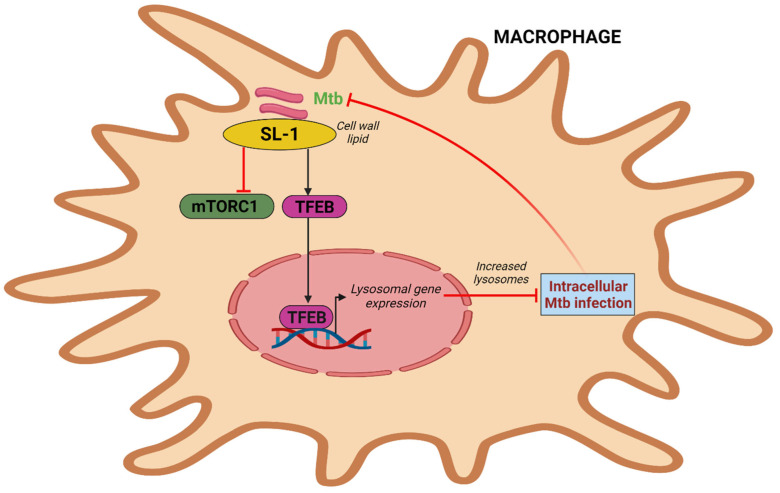
Intracellular Mtb simulates lysosome biogenesis via sulfolipid-1. Mtb sulfolipid-1 suppresses the activity of mTORC1 and causes the translocation of TFEB into the nucleus, which induces lysosomal gene expression and promotes lysosome biogenesis and subsequently Mtb clearance.

The most prevalent sulfated glycolipid in mycobacteria’s outer membrane and cell wall, sulfolipid-1 makes up to 1–2% of the dry weight of the cell wall but is only present in pathogenic mycobacteria [71]. Multiple mechanisms govern sulfolipid-1 synthesis, which has been shown to increase during infection in murine and human macrophages [72,73]. According to a recent study, pure Mtb sulfolipid-1 influences the kinetics of phagosome trafficking in macrophages and stimulates lysosomal biogenesis in host cells without regard to the cell type. Sulfolipid-1 works by preventing mTOR activity, which then causes TFEB to translocate into the nucleus and activate the expression of genes involved in lysosomal biogenesis (Figure 3). Human macrophages infected with Mtbpks2, a mutant that does not produce Sulfolipid-1 and lacks polyketide synthase 2, exhibit reduced lysosomal rewiring. Reduced bacterial interaction with lysosomes is caused by altered lysosomal activities, which is correlated with higher intracellular Mtb survival. In contrast, the mutant overproducing Sulfolipid-1 exhibits limited bacterial survival. The fact that mutants overproducing acetylated sulfated glycolipid (AC4SGL) are unable to stop phagosome maturation and are easily transported to lysosomes supports this as well [74]. To determine whether Mtb harbors a lipid that is beneficial to the host or whether targeting sulfolipid-1 could be a host-directed method to combat Mtb infection, more research will be needed [75].

## 6. Conclusions and Future Perspective

By modulating the expression of genes involved in autophagy and lysosomal biogenesis, the TFEB is a crucial transcriptional regulator that regulates Mtb intracellular clearance as well as energy consumption during hunger or stress. Essentially, the TFEB takes part in physiological processes like hunger and cellular responses to Mtb infection. Understanding how cells react to environmental stress or famine conditions, like food shortage, has been largely dependent on the action of the TFEB. Although it has been demonstrated that several transcription factors can trigger autophagy, TFEB seems to play a much more significant role because it regulates genes that are involved in numerous critical stages of the autophagic pathways, such as the formation of autophagosomes, the fusion of autophagosomes and lysosomes, and the degradation of the autophagosomal content. A thorough knowledge of the different extracellular and intracellular stimuli that control and customize an ideal response to stimulate TFEB-dependent lysosome formation and autophagy genes followed by clearance of engulfed cargo is still required, despite the existence of several studies. The TFEB is a therapeutic medication target for many human disorders that are linked to autophagic or lysosomal dysfunction and the buildup of toxic aggregates because of its role in intracellular clearance pathways. Several disease models, including Mtb, have already had success with the treatment approach of inducing the TFEB activation [76]. Compounds that target the TFEB may have fewer pleiotropic and undesirable side effects and may therefore be a possible target to treat tuberculosis in conjunction with fasting and stress circumstances like dietary deficits. This is because the TFEB directly regulates effector functions that induce autophagy. The TFEB could be developed as a viable host-directed target because it is differently and tightly controlled by many host variables during both infection and stress situations. However, it should be noted that alteration of the TFEB activity either induction or suppression may result in different biological outputs that may be further affected by many biological or physical factors. Also, TFEB signaling is reported to have heterogeneity in different cell types that further need to be investigated in different subsets of Mtb-infected macrophages. In consideration of the differential activity of the TFEB, it is important to coordinate its activity judiciously along with substantial information. Overall, the TFEB could be a potential target to eradicate Mtb, however, further detailed studies are needed considering differential regulation of the TFEB-induced autophagy in Mtb infection and starvation.

## Data Availability

Not applicable.
